# Organoids and tissue/organ chips

**DOI:** 10.1186/s13287-024-03859-1

**Published:** 2024-08-05

**Authors:** Graham Sean, Albert J. Banes, Rajashekhar Gangaraju

**Affiliations:** 1 Department of Ophthalmology, University of Tennessee Health Science Center, 930 Madison Ave, Suite 768, Memphis, TN 38163 USA; 2 Joint Department of Biomedical Engineering, University of North Carolina at Chapel Hill, and North Carolina State University, Chapel Hill, NC USA; 3 Flexcell International Corporation, Burlington, NC USA; 4 Department of Ophthalmology, Anatomy & Neurobiology, University of Tennessee Health Science Center, 930 Madison Ave, Suite 768, Memphis, TN 38163 USA

Organoids and spheroids are three-dimensional (3D) cell culture models that mimic the organization and functionality of specific organs and tissues. Derived from pluripotent stem cells, iPSCs, or adult tissue stem cells, they can self-assemble and differentiate into various cell types [1]. Complementing these advances, microfluidic tissue and organ chips have revolutionized the recreation of human organ complexity by incorporating multiple cell types, nutrient and growth factor supplies, and physiological cues, including nutrient infusion, geometric configuration, substrate stiffness and mechanical and electrical stimulation [2]. Integrating organoids with tissue and organ chips has yielded more realistic models for drug discovery, toxicity testing, diagnostics, therapeutics, and personalized medicine, effectively bridging the gap between in vitro experimentation and clinical applications [2]. This synergy enhances the predictive power of preclinical studies. It facilitates the development of tailored therapeutic interventions, advancing the field of regenerative medicine and offering new hope for treating complex diseases. Although stem cell constructs have demonstrated significant potential in modeling diseases, understanding pathological mechanisms, and developing new therapies, several challenges still exist, including variability in cell lineage and differentiation, scalability, and functional integration, which need to be addressed to realize potential clinical applications fully [3].

This thematic collection, “Organoids and Tissue/Organ Chips,” aimed to seek out research articles deepening our understanding of the utility of pluripotent stem cells or adult tissue stem cells as functional units of organoids and tissue/organ chips. In pursuit of this goal, we have gathered publications of many fascinating articles, including original research and reviews. These articles provide unique perspectives on problems faced in the development of organoids and spheroids, as well as their potential therapeutic implications, and elaborate on the future directions of the field. We believe the articles published in this collection have advanced the field and added knowledge to increase our understanding of organoids and spheroids and the questions surrounding their clinical applications.

Alongside therapeutic advances, the demand for organ substitutes has significantly increased. In response to this rising demand, Zhan et al. [4] reviewed fabrication and assembly techniques for creating functional tissue and organ substitutes. This article examines the dichotomy between top-down and bottom-up strategies [5–7]. Top-down processing consists of seeding cells onto biomaterial scaffolds and stimulating cell behavior and extracellular matrix production through growth factor supplementation [8]. However, this method often faces pitfalls of low cell density and uneven cell distribution, making recreation of tissue and organ microstructure difficult [8]. In contrast, the bottom-up approach in tissue engineering focuses on replicating living tissues by fabricating and assembling building blocks to enhance cell distribution, viability, vascularization, and integration in artificial tissues [8]. Techniques such as bioprinting, including inkjet, extrusion-based, and light processing methods, are recommended for shaping and positioning these blocks [9]. However, these methods primarily process biomaterials and indirectly impact cells, introducing some degree of randomness [9]. Bioprinting, a standout technology in bottom-up tissue engineering, enables the precise spatial organization of biomaterials and cells, enhancing tissue complexity and functionality [9]. Recent studies highlight bioprinting’s role in improving cell distribution, viability, and vascularization within artificial tissues, utilizing inkjet, extrusion-based, and light processing methods to create intricate 3D structures [5, 10, 11]. The authors impress upon the reader the potential of bioprinting to improve cell distribution and vascularization, noting the challenges of developing mechanically robust bio-inks and achieving adequate scalability.

Further work is needed in refining bioprinting techniques to overcome current limitations and meet clinical demands. Advances in gene editing to enhance cell functions, coupled with innovations in bioink development, especially with ligand binding and stiffness tuning and bioprinting speed, promise to bolster tissue engineering capabilities [12, 13]. The evolution towards 4D bioprinting, integrating time as a variable, holds the potential for creating dynamic tissues that respond to physiological cues [14, 15]. Additionally, as discussed by the authors, microfluidic systems like organ-on-a-chip (OoC) devices provide another versatile tool for studying disease and testing drugs. OoC devices recapitulate critical aspects of organ physiology, simulate complex interactions between and among different tissue types, and support co-culture or multi-organ systems [16]. As researchers continue to innovate, integrating mechanical improvements and biological insights will be crucial in achieving functional artificial tissues that replicate the complexity of native organs. This will ultimately advance patient care and therapeutic outcomes in regenerative medicine.

Similarly focused on furthering our understanding of standard and scalable production of hematopoietic organoids, Chen et al. [17] explored the creation of human hematopoietic organoids using Gelatin-methacryloyl (GelMA) hydrogels. These organoids mimic the bone marrow microenvironment and exhibit enhanced functionality, crucial for modeling diseases such as radiation injury and bone marrow fibrosis [18, 19]. The study’s innovative approach of incorporating niche-related cells within GelMA hydrogels supports robust hematopoietic cell proliferation and multilineage differentiation, mirroring physiological conditions more accurately than traditional 2D cultures. Importantly, the organoids they developed demonstrated responsiveness to radiation-induced injury, showing elevated ROS levels and DNA damage markers. Conversely, treatment of these models with G-CSF (granulocyte colony stimulating factor) post-irradiation, led to a decrease in apoptosis and enhanced hematopoietic cell recovery. These findings highlight their potential for therapeutic development and drug testing in radiation-induced bone marrow damage. This research paves the way for scalable production methods of these organoids, broadening their applications in studying disease mechanisms and evaluating novel therapeutic interventions. Hurdles remain in refining the fidelity of organoid models to complex in vivo hematopoietic environments and improving their scalability for widespread use. Methodological advances in optimizing the composition of GelMA-based scaffolds and enhancing the integration of niche cells will be critical.

This collection also boasts in-depth evaluations of various stem cell model types and organoid generation. Among those discussing such advances, Naderi-Meshkin et al. [20] highlighted the development of iPSC-derived vascular organoids, which more accurately model vascular complications associated with diabetes. Despite challenges in organoid size and composition, these models offer valuable insights into vascular biology and disease mechanisms, paving the way for personalized medicine applications. iPSC-derived vascular organoids represent a significant leap forward in creating functional blood vessels that mimic disease conditions such as diabetic vasculopathy [21]. These models provide valuable insights into the complex interactions between ECs and mural cells within the vasculature, insights which are crucial for understanding disease progression and testing therapeutic interventions. By integrating stem cell technologies with advanced bioengineering methods like microfluidics and adjustable matrices, researchers can enhance the reproducibility and maturity of these organoids. This integration not only improves disease modeling capabilities but also opens new avenues for personalized medicine, where patient-specific iPSC-derived vascular organoids could be used for drug screening and developing targeted therapies. Continued advances in this field hold promise for creating organ-specific vascular models that accurately replicate disease states, offering a powerful tool for studying cardiovascular diseases and advancing regenerative medicine approaches.

Pecksen et al. [22] take a similar approach, focused on improving the maturity and functionality of induced pluripotent stem cell (iPSC)-derived kidney organoids by incorporating immune cells during differentiation. One method to enhance kidney organoid differentiation in vitro involves incorporating additional cell types crucial for kidney development in vivo, such as macrophages [23] and vascular cells [24, 25]. This study focuses on enhancing the maturity and functionality of iPSC-derived kidney organoids by incorporating monocytes or iPSC-derived macrophages during differentiation. CHIR 99,021 (CHIR) is a glycogen synthase kinase-3 inhibitor and was used to induce mesodermal differentiation in IPSCs as the initial step for generating kidney organoids. However, a notable limitation of CHIR is its simultaneous induction of apoptosis in IPSCs. The researchers successfully counteracted this apoptosis with rapamycin and co-cultured IPSCs with macrophages. Moreover, the co-culture with monocytes significantly promoted the expression of renal differentiation markers and improved organoid development, primarily through the release of extracellular vesicles. This approach highlights the potential of combining cell types important for kidney organogenesis and using autophagy-modulating agents to enhance the differentiation and survival of kidney organoids.

Additionally, the research highlights that co-culturing iPSCs with these immune cells improves organoid survival and differentiation through mechanisms involving extracellular vesicles (EVs) and autophagy induction. Specifically, monocyte-derived EVs play a pivotal role in enhancing iPSC survival and promoting renal differentiation marker expression, demonstrating their potential to refine disease modeling and drug testing applications in nephrology. By mitigating CHIR-induced apoptosis and oxidative stress in iPSCs, this approach not only enhances organoid quality but also underscores the crucial role of autophagy in optimizing iPSC-based models for kidney research.

In another exciting look into the use of organoids serving as a model for studying drug interactions, Inui et al. [26] set out to overcome problems in current drug discovery research conducted in small intestinal tissues derived from experimental animals and Caco-2 cells. These issues include species differences [27] and lower expression levels of drug-metabolizing enzymes compared to human cells [28]. This group demonstrated the development of intestinal organoids from human-induced pluripotent stem cell-derived enterocyte-like cells (ELCs), representing a significant breakthrough in pharmacokinetic research. This study achieved robust differentiation of ELCs into functional intestinal organoids, followed by the optimization of a monolayer culture protocol (ELC-org-mono) that closely mimics the structural and functional characteristics of native human small intestine cells. Gene expression analyses revealed that ELC-org-mono exhibited comparable levels of intestinal markers to native tissue and significantly enhanced expression of crucial drug-metabolizing enzymes (CYP3A4, UGT1A1) and transporters (BCRP, PEPT1). This advance is pivotal for overcoming the limitations of current models, such as species differences and inadequate enzyme expression, thereby providing a reliable platform for studying drug absorption, metabolism, and transport in vitro.

Delivering further insight into the applications of organoids, Wang et al. [29] investigated chlorpromazine-induced bile duct injury using advanced in vitro cholangiocyte cultures. This research is motivated by the need to understand the mechanisms of drug-induced bile duct injury (DILI), specifically related to chlorpromazine (CPZ), a known inducer of cholestasis [30] and oxidative stress [31]. The experiments evaluated the expression of biliary transporters, oxidative stress markers, barrier function integrity, and inflammatory responses in cholangiocyte-like cell organoids (CLCOs). The study also investigates the impact of CPZ on cholangiocyte function under cholestatic and non-cholestatic conditions using CLCOs. The findings revealed that CPZ exposure altered the expression of several key transporters involved in bile acid transport, such as SLC51A, ABCB1, ABCC3, and SLC51B, suggesting a disruption in bile acid homeostasis.

Additionally, CPZ increased reactive oxygen species (ROS) production and depleted glutathione levels, indicating significant oxidative stress. Although CPZ alone didn’t trigger inflammation, it showed a synergistic effect when combined with TNFα. Furthermore, CPZ was found to compromise the integrity of the bile duct epithelial barrier by reducing the expression of tight junction proteins and E-cadherin, with more pronounced effects under cholestatic conditions. The findings of this study underscore the complex interplay between CPZ and bile acid transport, oxidative stress, barrier function, and inflammation, providing valuable insights into the mechanisms of CPZ-induced bile duct injury. The study also demonstrates the potential of CLCOs as an in vitro model for studying DILI and for use in preclinical drug safety testing.

In conclusion, this collection offers new insights and novel data for understanding the early damage or pathological mechanisms involved in various diseases. The articles published provide a wealth of knowledge regarding early assessment, pathological grading, and therapeutic implications of organoids and spheroids. The editors believe this set of articles will enhance clinical understanding in the field and invite further interdisciplinary research toward early assessment and development of treatment strategies.

Future research should focus on refining these models to improve reproducibility and functionality. Integrating supportive cell types, optimizing differentiation protocols, developing vascularization and innervation and advancing bioprinting techniques will be crucial. Additionally, exploring personalized medicine applications and developing targeted therapies based on these models hold significant promise for advancing clinical outcomes in regenerative medicine. The incorporation of biomechanical stimuli such as mechanical and electrical stimulation and substrate stiffness control should be explored to further simulate a native environment in developing OOC or microphysiological systems, which is vital to fully replicating in vivo physiology in health and disease [32, 33]. Finally, ethical and regulatory challenges, including but not limited to the use of human cells and the potential for creating complex, sentient responsive tissue, raise ethical questions that must be addressed. Similarly, regulatory challenges will be raised in standardizing and approving organoid-based methods for clinical and pharmaceutical applications. By continuing to address these challenges and leveraging new technologies, organoid research, and tissue-organ chips can achieve greater breakthroughs, ultimately enhancing our ability to treat a wide range of diseases and improving patient care.
